# The Effects of *BRCA1* and *BRCA2* Promoter Methylation on Clinicopathological Characteristics and Clinical Outcomes in HGSOC

**DOI:** 10.3390/cells15030277

**Published:** 2026-02-01

**Authors:** Katarina Živić, Ivana Boljević, Milica Nedeljković, Milana Matović, Radmila Janković, Miljana Tanić

**Affiliations:** 1 Experimental Oncology Department, Institute for Oncology and Radiology of Serbia, 11040 Belgrade, Serbia; db062020@student.chem.bg.ac.rs (K.Ž.);; 2 Clinic for Medical Oncology, Department for Breast Tumors and Gynecologic Malignances, Institute for Oncology and Radiology of Serbia, 11000 Belgrade, Serbia; 3 Medical Genomics, Cancer Biology Department, UCL Cancer Institute, University College London, London WC1E 6DD, UK

**Keywords:** high-grade serous ovarian cancer, methylation-specific PCR, promoter region, *BRCA1/2* genes

## Abstract

Ovarian cancer is a highly lethal disease. Tumors with a deficiency in the homologous recombination repair pathway (HRD) resulting from mutations in *BRCA1*/*2* genes have a favorable response to platinum-based chemotherapy and targeted therapy with PARP inhibitors (PARPi) mediated by synthetic lethality. Promoter methylation of *BRCA1/2* genes was previously associated with HRD, but little is known about whether it translates to clinical benefit. Here, we evaluated the prevalence of *BRCA1/2* promoter methylation in HGSOC patients from Serbia and examined their clinicopathological characteristics and the effect on progression-free and overall survival. Using methylation-specific PCR, we screened for hypermethylation in the promoter region of *BRCA1/2* genes in a cohort of 244 patients. We found fully methylated *BRCA1* and *BRCA2* promoter in 4.1% and 0.45% of patients, and 23.36% and 11.21% intermediately methylated cases, respectively. Full *BRCA1/2* promoter methylation was significantly associated with younger age of onset (55 and 58 years, respectively) compared to *BRCA1/2*-mutated cases, suggestive of BRCAness phenotype. However, in the exploratory analysis of 68 patients with clinical follow-up, we did not find a strong survival advantage for *BRCA1/2* methylated over *BRCA1/2*-intact cases, yet more moderate effects cannot be ruled out due to the cohort size.

## 1. Introduction

Ovarian cancer (OC) remains the most lethal gynecological cancer, and Europe has the highest age-standardized incidence in the world of 6.6 [1]. This disease is typically presented in advanced stages, due to limited screening techniques and the absence of specific symptoms [2]. Ovarian cancer is a heterogeneous disease with several histological subtypes that harbor different molecular characteristics [2]. Epithelial ovarian cancer (EOC) is the most common one, with the high-grade serous subtype representing the vast majority of all cases [3,4].

The influence of germline and somatic mutations in *BRCA1* and *BRCA2* tumor suppressor genes is well established in the origin of ovarian cancer. The lifetime risk of developing OC is 28–66% in patients harboring *BRCA1* alterations, and 16–27% in those carrying *BRCA2* mutations [5,6,7]. BRCA1 and BRCA2 proteins are necessary for the repair of double-strand DNA breaks (DSBs) and interstrand cross-links (ICLs) by homologous recombination [8]. Homologous recombination cannot be performed in the cells with mutated *BRCA1* or *BRCA2*, and DSBs are repaired through alternative error-prone mechanisms, which may cause genetic instability in that cell and malignant transformation. DSBs can be repaired in mammalian cells by non-homologous end joining (NHEJ), which is potentially error-prone because nucleotide changes are accommodated at the rejoining sites, or single-strand annealing (SSA), where homology is between extended stretches of single-stranded DNA (ssDNA) [8]. The resulting homologous recombination deficiency (HRD) is manifested by a specific “genomic scar” characterized by the loss of heterozygosity (LOH), large-scale transitions (LSTs), and telomeric allelic imbalance TAI.

**Figure 1:**
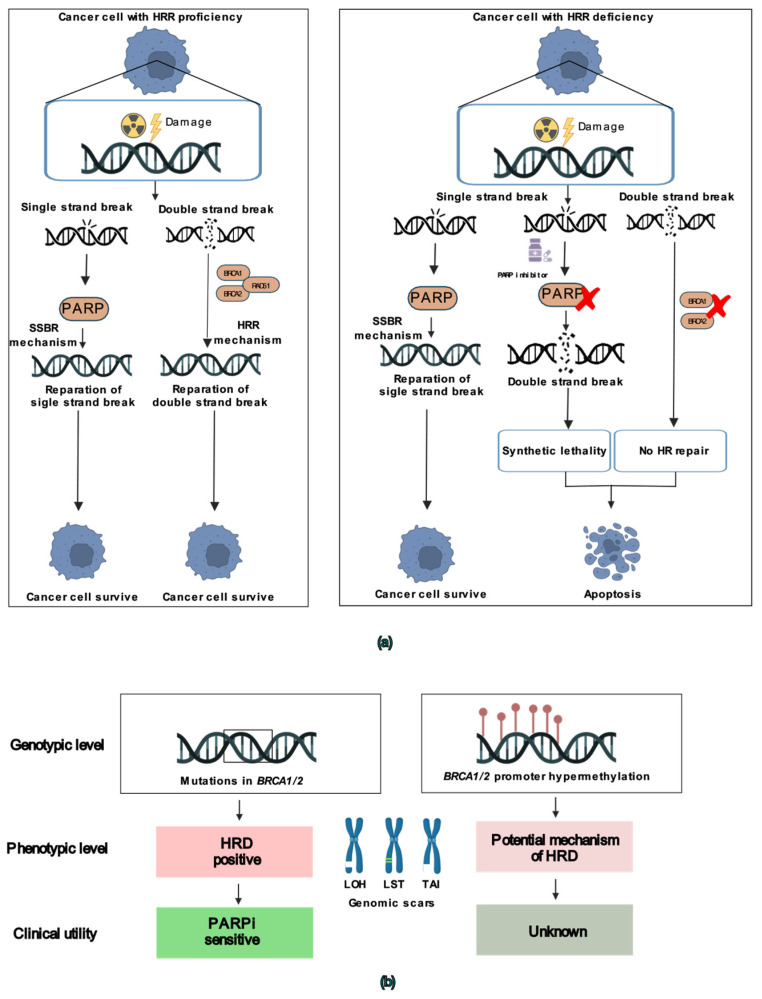
Representation of PARP protein function and HRD causality. (**a**) PARP-mediated repair of single-strand DNA breaks (SSBs), in which PARP detects DNA damage and recruits repair proteins to restore DNA integrity (left). Inhibition or loss of PARP activity leads to the accumulation of unrepaired SSBs, and subsequent double-strand DNA breaks (DSBs), which leads to HRD phenotype (right); (**b**) Genetic and epigenetic mechanisms underlying homologous recombination deficiency (HRD).

Patients with *BRCA1/2* mutations show better response to platinum-based chemotherapy generating ICLs, as well as to targeted treatment with poly (ADP-ribose) polymerase inhibitors (PARPis) by the mechanism of synthetic lethality (**Figure 1a**) [9,10]. The prevalence of germline and somatic mutations in *BRCA1/2* genes varies, ranging between 17 and 25%, while HRD phenotype was shown to be present in approximately half of OC patients and was recently introduced as an independent biomarker for therapy with PARPi [11,12].

Beyond *BRCA1/2* mutations, mutations in other homologous recombination repair genes or silencing by epigenetic mechanisms may also cause HRD. Hypermethylation of CpG islands in regulatory regions of tumor suppressor genes is already established for many malignancies [13]. In ovarian cancer, several studies reported *BRCA1* promoter hypermethylation in ~10% of cases, and more rarely, *BRCA2* promoter hypermethylation [11]. As *BRCA1/2* promoter methylation was shown to be associated with BRCAness-like phenotype and to be a 100% predictor of HRD [14], it was postulated that the treatment with platinum-based therapy and PARPi would be as successful in tumors with this type of epigenetic alteration and could be used as an independent biomarker. However, there is a lack of concordance among studies of clinical benefit conferred by a potentially reversable epigenetic inactivation of *BRCA1/2*.

The prevalence of hereditary *BRCA1/2* mutations is known to be population-specific, ranging from 5 to 10% in Western European populations to as high as 26–50% in Slavic and Asian populations [15,16,17]. However, little is known regarding the prevalence of epigenetic alterations among non-western European populations. In this study, we aimed to evaluate the prevalence of *BRCA1/2* promoter methylation in a cohort of ovarian cancer patients who underwent tumor *BRCA1/2* mutation testing at the Institute for Oncology and Radiology of Serbia (IORS). Additionally, expression analysis was performed to provide insight into gene silencing, as well as the impact of different genetic and/or epigenetic alterations on the level of mRNA. In a subgroup of patients, we evaluated the impact of *BRCA1/2* promoter methylation on progression-free survival (PFS) and overall survival (OS) rate, as well as on different clinicopathological characteristics.

## 2. Materials and Methods

### 2.1. Patients and Samples

**Figure 2:**
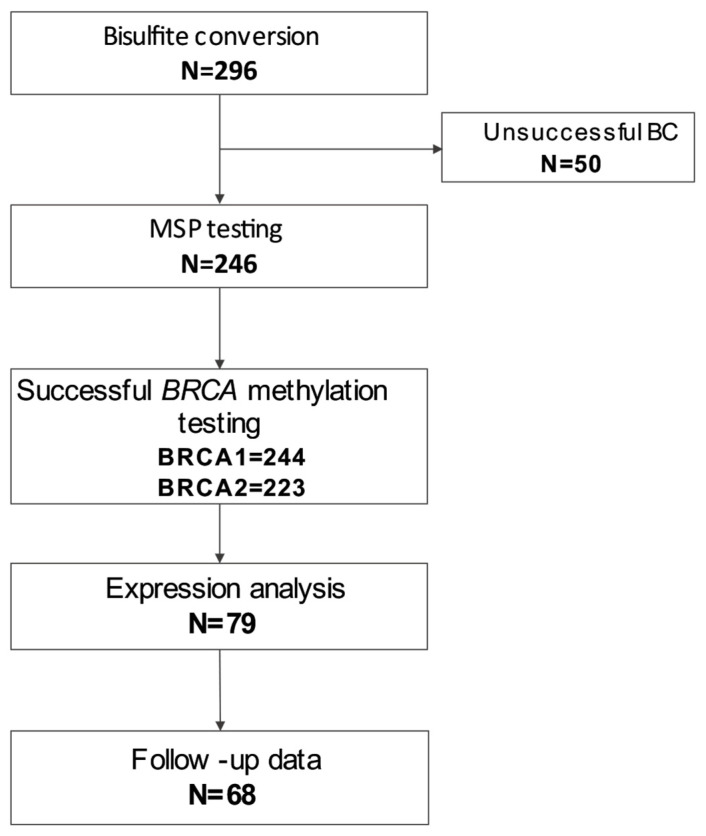
Flowchart describing the cases analyzed in this study.

The study group was a retrospective cohort drawn from consecutive ovarian cancer patients, unselected for family history, who underwent reflex *BRCA1/2* tumor testing at the Institute for Oncology and Radiology of Serbia (IORS) from 2019 to 2023. The eligibility criteria were present information about the *BRCA1/2* mutation status and enough isolated DNA for further methylation analysis. A total of 296 samples were selected for *BRCA1/2* promoter hypermethylation testing by methylation-specific PCR (MSP) from exceeding diagnostic FFPE tumor material. One group of 50 samples was excluded because of unsuccessful pretreatment of DNA prior to MSP; in total, 246 samples were tested by MSP. One subgroup of 79 methylation-tested samples, for which there was sufficient leftover FFPE blocks for RNA isolation, was used for testing the expression level of *BRCA1/2* genes by qRT-PCR technology. For a total of 68 patients treated at IORS, we were able to obtain clinicopathological data from the National Health Repository to be placed into an anonymized study database. The study was conducted in accordance with the Declaration of Helsinki and approved by the local Ethics Committee (No. 01-1/2023/702). In **Figure 2**, the flowchart of the study design is represented.

### 2.2. DNA and RNA Extraction

FFPE blocks obtained from primary surgery from women with serous ovarian cancer were used to extract DNA and RNA. Tumors were fixed and prepared by local pathology departments after material was gathered from patient surgeries conducted at IORS and/or medical facilities throughout Serbia. The tumor tissue percentage (TTC%) was determined by a resident pathologist, and the identified tumor-rich tissue areas ware macro-dissected. DNA was extracted using QIAamp FFPE Tissue Kit (QIAGEN, Hilden, Germany), according to the manufacturer’s instructions. In short, after the macro-dissection of FFPE blocks, xylol and absolute ethanol were used for deparaffinization, ATL buffer and proteinase K were used for lysis for 1 h at 56 °C and 1 h at 90 °C, and isolation and purification of DNA were performed in QIAamp MinElute columns. Elution volume was 50–100 µL of nuclease-free water. Quantification was performed using the Qubit HS/BR dsDNA kit. RNA material was extracted by RNeasy FFPE Kit (Qiagen, Hilden, Germany) according to the manufacturer’s recommendation. The deparaffinization procedure was performed with xylol and ethanol, PKD buffer and proteinase K, with incubation steps on 37 °C, 56 °C, and 80 °C, after which RNA was extracted on columns and eluted with 17 µL of nuclease-free water. The derived RNA was quantified using the BioSpec-nano Spectrophotometer (Shimadzu Biotech, Kyoto, Japan).

### 2.3. Bisulfite Conversion

The conversion of unmethylated cytosine was performed by bisulfite treatment [18]. Bisulfite conversion and subsequent purification was performed using one of the available kits—EZ-96 DNA Methylation-Lightning™ MagPrep, EZ-96 DNA Methylation-Direct MagPrep and EZ-96 DNA Methylation-Direct (Zymo Research, Irvine, CA, USA), according to the respective kit protocols. The success of bisulfite conversion was confirmed by the amplification of samples with primers for housekeeping gene *Actinβ* (FW: 5′-TGGTGATGGAGGAGGTTTAGTAAGT-3′; REV: 5′-AACCAATAAAACCTACTCCTCCCTTAA-3′) on Light Cycler 480 Instrument (Roche, Basel, Switzerland) machine under the following conditions: one cycle of denaturation at 95 °C for 10 min, followed by 40 cycles of the 15 s at 95 °C and 1 min at 60 °C, using Power SYBR Green Master Mix (Applied Biosystems Thermo Fisher Scientific, Waltham, MA, USA), or by PCR protocol using AmpliTaq Gold 360 master mix (AppliedBiosystems, Thermo Fisher Scientific, Waltham, MA, USA) and subsequent visualization on 2% agarose gel electrophoresis.

### 2.4. Methylation Status Analysis

A methylation-specific PCR (MSP) reaction was used to detect the methylation profile. For this purpose, we utilized two sets of primers for each of the tested genes. The unmethylated DNA was amplified using one set of primers, and the methylated DNA was amplified using another set of primers. All primers were extracted from the literature [19]. For *BRCA1* gene methylation analysis, primer pairs were as follows: methylated forward 5′-TCGTGGTAACGGAAAAGCGC-3′, methylated reverse 5′-AAATCTCAACGAACTC ACGCCG-3′, unmethylated forward 5′-TGGTTTTTGTGGTAATGGAAAAGTGT-3′, unmethylated reverse 5′-CAAAAAATCTCAACAAACTCACACCA-3′ [20]; and for *BRCA2* gene methylation analysis: methylated forward 5′-GCGGTAGAGGCGGAGTC-3′, methylated reverse 5′-CGAAATAAACTAACAAAAACCG-3′, unmethylated forward 5′-ATTAGGTGGTAGAGGTGGAGTT-3′, unmethylated reverse 5′-CCAAAATAAACTAA CAAAAACCA-3′ [21]. The PCR amplification was performed with bisulfite-treated DNA as a template, using commercial PCR master mix AmpliTaq Gold 360 (AppliedBiosystems, Thermo Fisher Scientific, Waltham, MA, USA), and cycling conditions were as follows: denaturation at 95 °C for 10 min, followed by 40 cycles of the 30s at 95 °C, 30 s at 60 °C, 45 s at 72 °C, and one cycle of final elongation of 7 min at 72 °C for *BRCA2* analysis; for *BRCA1*, we used the program and protocol previously described by Ben Gacem et al. [19]. Commercial positive (fully methylated) and negative (fully unmethylated) controls were tested in the same way alongside the samples (Human Methylated and Unmethylated DNA Set, Zymo Research, Irvine, CA, USA). PCR products were visualized on 2% agarose gel by electrophoresis or with high sense and/or 1000DNA chips on Agilent 2100 Bioanalyzer instrument (Agilent, Santa Clara, CA, USA). Samples were referred to as FM—fully methylated—if there was only one band in the agarose gel in the well with the PCR product with the primers for the methylated sequence. Those with only one band in the agarose gel in the well with the PCR product with the primers for the unmethylated sequence were referred to as UM—unmethylated. Samples with both bands were classified as IM—intermediately methylated—which may represent either the allele-specific methylation in tumor samples, subclonal fully methylated tumor tissue, or the clonal fully methylated tumor cells confounded with unmethylated normal stroma and/or immune infiltrate.

### 2.5. Real-Time PCR

To detect the expression level of *BRCA1/2* genes, we performed a quantitative real-time PCR reaction. Reverse transcription was performed using High-Capacity cDNA Reverse Transcription Kit (Applied Biosystems, Vilnius, Lithuania). Expression measuring was performed by quantitative real-time PCR on Light cycler 480 Instrument (Roche, Basel, Switzerland) using Power SYBR Green Master Mix (Applied Biosystems, Thermo Fisher Scientific, Waltham, MA, USA). For this reaction, we used primers that were previously successfully used and were retrieved from PrimerBank (https://pga.mgh.harvard.edu/primerbank/ (accessed on 16 June 2023)). Primer pairs were as follows: for *BRCA1* forward 5′-ACCTTGGAACTGTGAGAACTCT-3′, reverse 5′-TCTTGATCTCCCACACTGCAATA-3′; for *BRCA2* forward 5′-TGCCTGAAAACCAGATGACTATC-3′, reverse 5′-AGGCCAGCAAACTTCCGTTTA-3′; housekeeping *GAPDH* gene forward 5′-GACAGTCAGCCGCATCTTCT-3′, reverse 5′-GCGCCCAATACGACCAAATC-3′. PCR conditions included initial denaturation at 95 °C for 2 min, followed by 45 cycles of 15 s at 95 °C and 1 min at 60 °C. Missing Ct values were overwritten with the maximum number of cycles in the reaction. Further, ΔCt was calculated relative to GAPDH, as the subtraction of Ct values of the gene of interest and Ct of GAPDH. ΔΔCt was calculated as the subtraction of ΔCt and the median ΔCt value for the gene. Finally, fold change was calculated as a negative exponential of the ΔΔCt value, according to the formula 2^−ΔΔCt^, and used in statistical analysis.

### 2.6. Statistics

Descriptive statistics were used to ascertain the patients’ clinical and demographic characteristics. Overall survival (OS) was computed from the date of the initial diagnosis to the date of death or the loss of follow-up. The period between the initial diagnosis date and the date of recurrence or the most recent follow-up was used to compute progression-free survival (PFS). Progression-free interval (PFI) was calculated from the date of the last chemotherapy cycle to the date of progression/recurrence or loss of follow-up. Using Fisher’s exact test, we examined the association of promoter methylation and mutation of the *BRCA1* and *BRCA2* genes, as well as the association with clinicopathological data. The Shapiro–Wilk test was employed for determining the normality of distribution, and the differences in age distribution across groups were tested by the Kruskal–Wallis test, with the post hoc Dunn test. We used the same Kruskal–Wallis test to assess whether expression levels differ within the methylation and/or mutation groups. The Kaplan–Meier technique was used to plot survival functions and the log-rank test was used to test for differences in survival between groups. Cox proportional hazard regression models were used to perform multivariable analysis of OS, adjusting for mutation status, age, patient group, and therapy approach using the “survival” version 3.8-3 R package. Post hoc power analysis was performed using the “powerSurvEpi” version 0.1.5. R package. Statistical analysis was performed using RStudio (version: 4.3.1). Two-tailed *p*-values less than 0.05 were considered statistically significant.

## 3. Results

### 3.1. Patient Characteristics

The cohort tested for hypermethylation consisted of both newly diagnosed (ND) and relapsed (R) cases of HGSOC patients due to evolving clinical criteria used to refer patients for *BRCA1/2* tumor mutation testing as companion diagnostics for PARPi therapy. *BRCA1/2* methylation testing was always performed on FFPE blocks obtained during primary cytoreductive surgery or biopsy prior to first-line treatment, thus providing the baseline methylation status of these tumors.

The cohort of 296 samples selected for *BRCA1/2* promoter hypermethylation testing consisted of 186 relapsed and 109 newly diagnosed cases of ovarian cancer. After bisulfite conversion, a total of 246 successfully converted samples were tested by MSP. The tested group consisted of 190 *BRCA1/2* benign (wild-type—WT), 21 *BRCA1* pathogenic/likely pathogenic, 21 *BRCA2* pathogenic/likely pathogenic, and 14 *BRCA1/2* VUS cases of ovarian cancer patients that were treated as WT. Two patients were excluded, as the MSP testing was unsuccessful for both *BRCA1* and *BRCA2*. The median age at testing was 62 (range: 17–86), and 61 at diagnosis (available for 150 patients—newly diagnosed and relapsed with the available information about disease history). Most cases (~90%) were presented in the advanced stage (FIGO category III and IV). Regarding the treatment approach, 58% of patients, whose clinicopathological data we were able to obtain, were treated with chemotherapy in combination with targeted maintenance therapy with either bevacizumab or olaparib. Response to platinum-based treatment was described as sensitive if more than 12 months passed from the last chemotherapy cycle to progression; it was described as partial if PFI was between 6 and 12 months; and patients with PFI < 6 months were resistant. Patients and clinical characteristics are presented in Table 1.

### 3.2. Prevalence of Promoter Hypermethylation in BRCA1/2 Genes

A total of 296 DNA samples were bisulfite converted. Bisulfite conversion treatment was not successful in 50 samples; hence, the hypermethylation of *BRCA1/2* promoter regions was tested in a total of 246 patients, with successful *BRCA1* hypermethylation testing in 244 (99.19%) samples and *BRCA2* hypermethylation testing in 223 (90.65%) samples.

**Figure 3:**
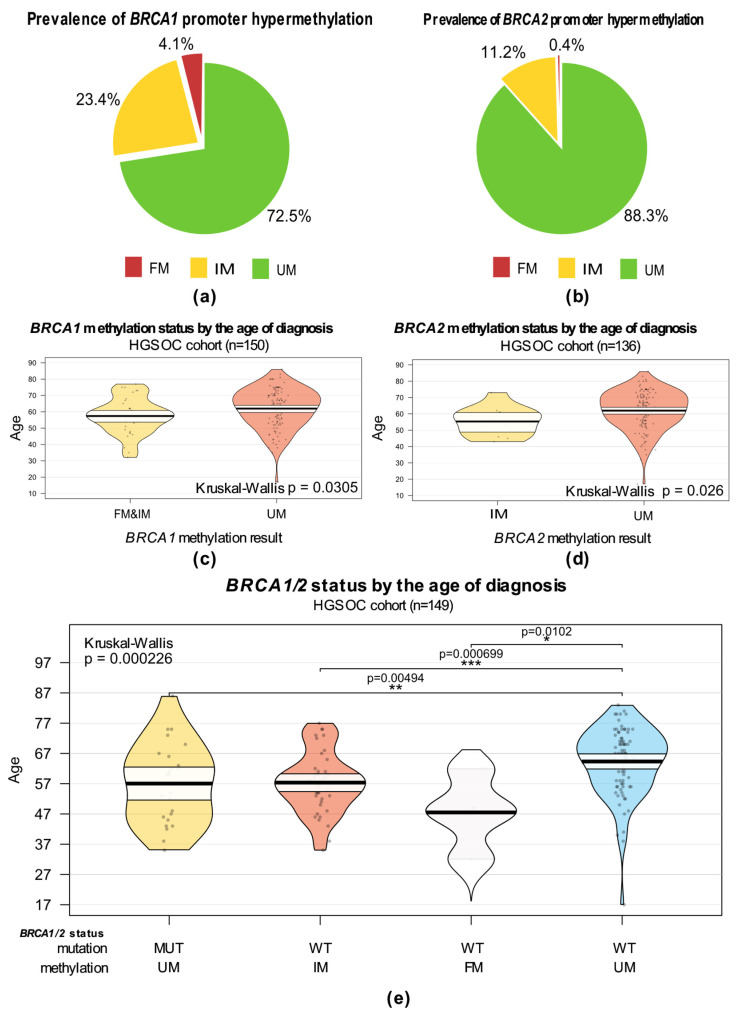
*BRCA1/2* promoter hypermethylation and clinical characteristics of HGSOC-tested cohort. (**a**) Prevalence of *BRCA1* promoter hypermethylation. (**b**) Prevalence of *BRCA2* promoter hypermethylation. (**c**) Pirate plot of age at diagnosis by *BRCA1* methylation status (n = 150). (**d**) Pirate plot of age at diagnosis by *BRCA2* methylation status (n = 136). (**e**) Pirate plot of age at diagnosis by *BRCA1/2* aberration status (n = 149); Kruskal–Wallis rank sum test with post hoc Dunn’s test, non-significant comparisons are not represented, *** represents *p* ≤ 0.001, ** represents *p* ≤ 0.01 and * represents *p* ≤ 0.05; FM—fully methylated; IM—intermediary methylated; UM—unmethylated; MUT—*BRCA1/2*-mutated.

We evaluated the percentage of patients who had a fully methylated (FM), intermediately methylated (IM), or unmethylated (UM) promoter region of the *BRCA1* and *BRCA2* genes based on the presence of a band in the agarose gel electropherogram after methylation-specific PCR (Supplementary Figure S1). While a fully methylated or unmethylated result implies the clonal homozygous methylation status of both alleles, the intermediate level of methylation reflects the presence of cells with both unmethylated and methylated CpGs in the promoter but does not provide information on the level of methylation, its origin, zygosity, or clonal architecture. Fully methylated promoter was found in a small number of samples; for *BRCA1* it was 4.1% of samples (10/244), and for *BRCA2* it was 0.45%, only in one sample (1/223). Intermediately methylated *BRCA1* and *BRCA2* promoters were found in 23.36% (57/244) and 11.21% (25/223) of samples, respectively. The rest of the samples were found to have fully unmethylated promoter: 72.5% (177/244) for *BRCA1* and 88.34% (197/223) for *BRCA2* (Figure 3a,b).

### 3.3. Epigenetic Alterations and Clinicopathological Characteristics

The clinicopathological features based on *BRCA1/2* methylation status in the tested cohort are represented in Table 2, and the ones based on overall *BRCA1/2* aberration status are presented in Supplementary Table S1.

The mean age of diagnosis was 62 for both *BRCA1* and *BRCA2* methylation-tested groups. By comparing the age distribution at diagnosis according to the methylation test result, shown in Figure 3c,d, we found that cases with methylated promoters of *BRCA1* or *BRCA2* genes are more common in younger patients (*p* = 0.026 and *p* = 0.0305, respectively). However, the difference lost its significance (*p* = 0.0618) when FM and IM cases are considered separately for *BRCA1* (Supplementary Figure S2a). However, the number of FM cases in *BRCA1* is very small for adequate comparison and for *BRCA2* we found hypermethylation in only one case aged 62 at testing. This case was included in the IM group for age statistics. We observed similarities in age distribution between *BRCA1/2*-mutated and *BRCA1/2* intermediately methylated cases, with *BRCA1/2* fully methylated patients being even younger (*p* = 0.000226) (Figure 3e). Distribution of age at testing for these groups is presented in Supplementary Figures S2 and S3.

Of 68 patients whose family history we were able to extract, 19 patients had a family history of either ovarian or breast cancer, and it was observed that the *BRCA1* gene is preferably unmethylated in this group of patients (two-sided Fisher’s test, *p* = 0.0277). However, no significant difference was found for *BRCA2* methylation status in relation to family history. There was no significant association of *BRCA1/2* methylation status and FIGO stage (Fisher’s exact test *p*-values of 0.573 and 0.147 for *BRCA1* and *BRCA2* methylation cohorts, respectively). We found a significant enrichment of tumors with both *BRCA1* and *BRCA2* baseline methylation among patients who had a relapse (*p* = 0.002 and *p* < 0.001, respectively). This group consisted exclusively of patients who had an initial good response to platinum chemotherapy, unlike the newly diagnosed group, which potentially consisted of both platinum-sensitive and platinum-resistant tumors.

### 3.4. Co-Occurrence Analysis of Genetic and Epigenetic BRCA1 and BRCA2 Inactivation

**Figure 4:**
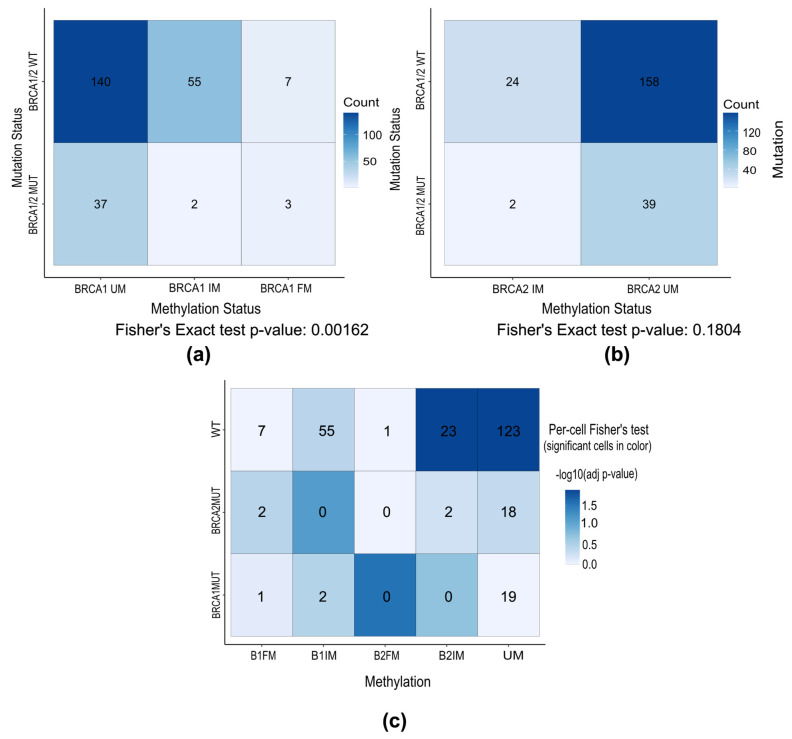
Association of *BRCA1/2* methylation and *BRCA1/2* mutations. (**a**) Heatmap of association of *BRCA1* hypermethylation and *BRCA1/2* alterations. Vast majority of cases are unmethylated and without any mutation in *BRCA1/2* genes. Additionally, 7 out of 10 fully methylated cases do not have mutation in either *BRCA1* or *BRCA2* gene. (**b**) Heatmap of association of *BRCA2* hypermethylation and *BRCA1/2* alterations. As there was only one case of *BRCA2* hypermethylation, this sample was added to IM group. S for *BRCA1* mutations in *BRCA1/2* are mutually exclusive to any methylation pattern. (**c**) Heatmap of association of genetic and epigenetic aberration in *BRCA1/2* with FDR adjusted *p*-values. Significant association was observed between *BRCA1/2* WT and *BRCA2* IM and *BRCA1/2* UM cases, as well as between *BRCA2* FM case and mutations in *BRCA1* gene. UM—unmethylated; FM—fully methylated; IM—intermediary methylated; B1—BRCA1; B2—BRCA2.

Using Fisher’s exact test, we tested for association between promoter methylation and mutation status in *BRCA1/2* genes to confirm if hypermethylation and mutations are mutually exclusive events. We found that *BRCA1* promoter methylation (FM and IM observed together as *BRCA1*_M) and *BRCA1/2* mutations are mutually exclusive (one-sided, alternative = “less”; *p* = 0.008; OR = 0.31). Association remains significant even when *BRCA1* methylation status is represented through all three categories as FM, IM, and UM (Fisher’s exact two-sided test; *p* = 0.0016), which is represented in Figure 4a. *BRCA1/2* mutation status is also represented in three categories: *BRCA1*_MUT, *BRCA2*_MUT, and WT (two-sided Fisher’s exact test, *p* = 0.0055). A fully methylated *BRCA1* promoter was found in two samples in co-occurrence with *BRCA1* VUS and pathogenic *BRCA1* alteration. And in two samples harboring pathogenic and likely pathogenic *BRCA2* variants, a fully methylated *BRCA1* promoter was detected. In three of these cases, *BRCA2* methylation status was described as intermediately methylated, and in *BRCA1*-mutated it was described as unmethylated.

**Figure 5:**
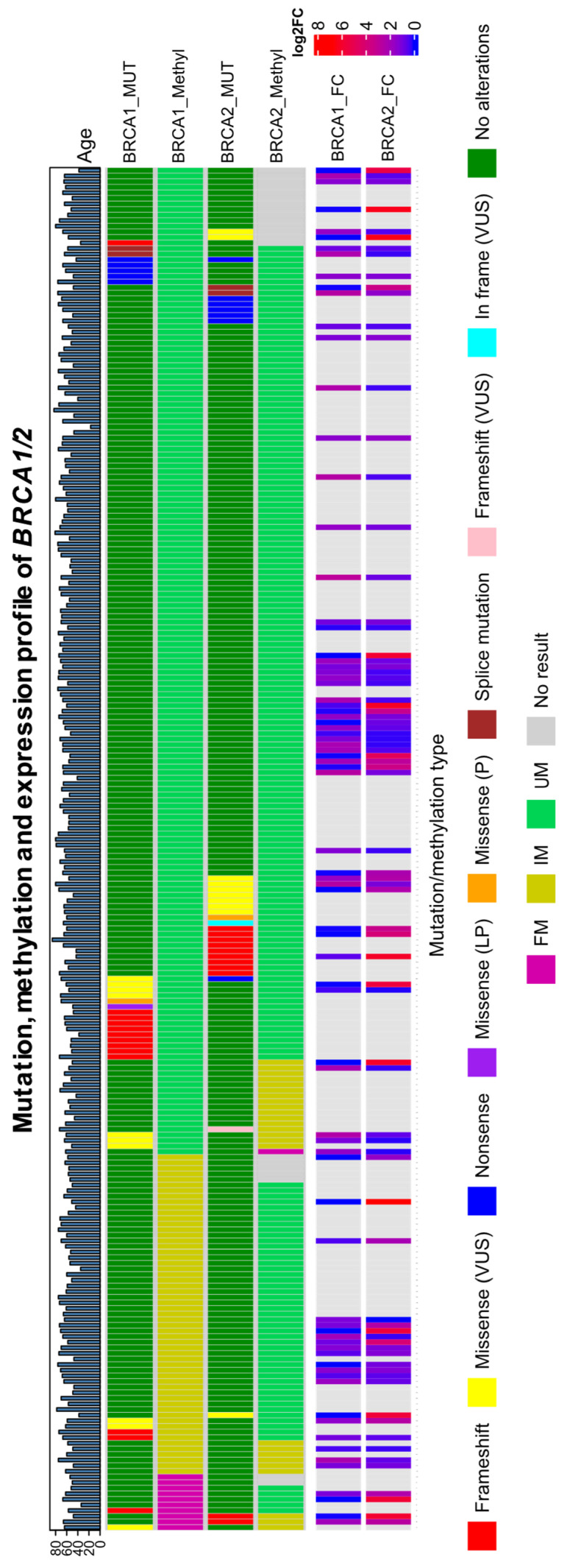
OncoPrint diagram of mutational and methylation frequencies in BRCA1/2 genes. Samples are sorted based on BRCA1 and BRCA2 promoter methylation status; UM—unmethylated; FM—fully methylated; IM—intermediary methylated; BRCA1_FC-BRCA1 fold change; BRCA2_FC-BRCA2 fold change; M—methylated; MUT—mutated.

No significant association was found between *BRCA2* promoter methylation and *BRCA1/2* mutation status (two-sided Fisher’s exact test, *p* = 0.1804 and *p* = 0.3711 when *BRCA1/2* mutations were represented in three categories) (Figure 4b), as well as between *BRCA1* and *BRCA2* methylation status (two-sided Fisher’s exact test, *p* = 0.1399). Most cases were wild-type and unmethylated for *BRCA1/2*. OncoPrint representation of all *BRCA1/2* aberrations found in patients in the HGSOC cohort from this study, as well as fold change rate of expression level, are shown in Figure 5.

### 3.5. Methylation Effects on Gene Expression

To verify if methylation and/or mutations have an impact on expression level, we performed quantitative RT-PCR of *BRCA1* and *BRCA2* mRNA. Expression level analysis was performed for 81 samples. Amplification of the housekeeping gene *GAPDH* was successful for 79 samples, for which we further performed statistical analysis.

**Figure 6:**
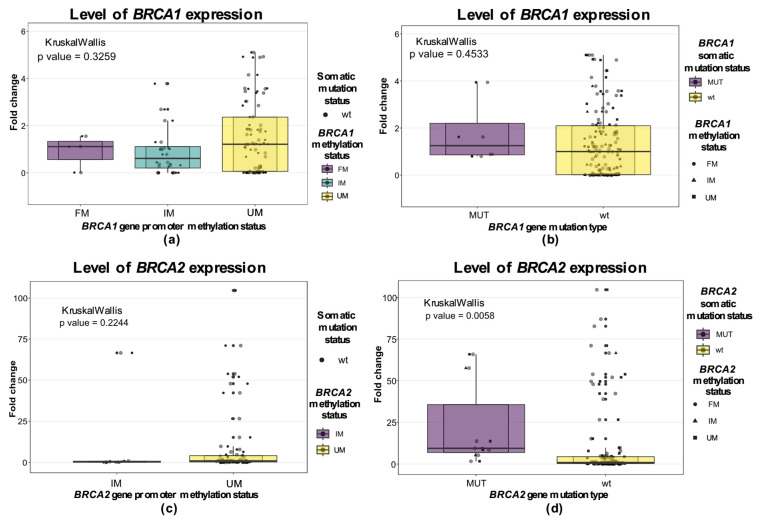
Boxplots of *BRCA1/2* expression level according to methylation or mutational status. (**a**) *BRCA1* expression level stratified by *BRCA1* methylation profile; (**b**) *BRCA1* expression level stratified by *BRCA1* mutation status; (**c**) *BRCA2* expression level stratified by *BRCA2* methylation level; (**d**) *BRCA2* expression level stratified by *BRCA2* mutation status. UM—unmethylated; FM—fully methylated; IM—intermediary methylated; MUT—mutated.

We analyzed the expression levels of the tested samples in relation to their methylation and mutation types. While we failed to discover a statistically significant difference between expression levels in relation to promoter methylation status for either of these two genes, there was a notably larger variance in *BRCA1* and *BRCA2* expression in the unmethylated groups (Figure 6). Observing *BRCA1/2* expression levels in relation to *BRCA1/2* mutation type, the results show a significantly higher expression level for *BRCA2* gene expression in the *BRCA2*-mutated group in comparison to WT samples (Kruskal–Wallis *p* = 0.0058; Figure 6d). However, the mutations in the *BRCA1* gene did not affect the expression of the same gene. Level of expression according to overall *BRCA1/2* aberration is presented in Supplementary Figure S4.

### 3.6. Exploratory Analysis of BRCA1/2 Promoter Methylation on Survival

From the whole *BRCA1/2* methylation cohort, for 68 patients, we had available clinicopathological follow-up data for exploratory survival analysis. We did not observe any selection bias for age, stage, or molecular features between the full cohort and the follow-up subset (Supplementary Table S3). The strong survival benefit of PARPi therapy observed for *BRCA1/2*-mutated HGSOC found in several clinical trials (SOLO1: HR = 0.55; PAOLA-1: HR = 0.60) motivated us to look for comparable effect sizes [22,23]. Our study was powered to detect only hazard ratios of under 0.5 due to the prevalence of the analyzed covariates (roughly 40% for combined FM and IM cases), at 80% power and alpha of 0.05. However, the study is underpowered (~40% power) due to large class imbalance to discern a comparable effect in FM vs. IM and FM cases and should be considered as exploratory. The range of follow-up was from 12 to 170 months, with a median follow-up of 42 months (3.5 years). We examined possible differences in OS and PFS rates according to *BRCA1/2* baseline methylation status in the *BRCA1/2* WT subgroup to test if any of these alterations have an impact on patient survival. We found no evidence of impact either for *BRCA1* methylation or for *BRCA2* methylation on progression-free survival (log-rank *p* = 0.87 and *p* = 0.55 for the *BRCA1* and *BRCA2* genes, respectively), or on overall survival (log-rank *p* = 0.34 and *p* = 0.94, respectively; Supplementary Figure S5).

**Figure 7:**
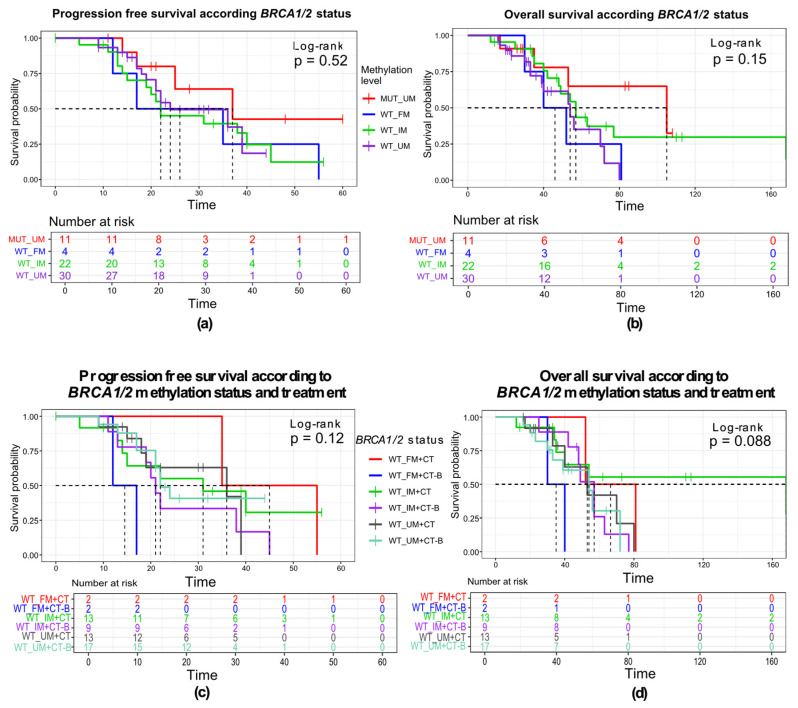
Kaplan–Meier survival curves according to BRCA1/2 status. (**a**) PFS according to BRCA1/2 aberration and treatment type. (**b**) OS according to BRCA1/2 aberration and treatment type. (**c**) PFS according to BRCA1/2 aberration type. (**d**) OS according to BRCA1/2 aberration type. OS = overall survival, PFS = progression-free survival; WT—wild-type; UM—unmethylated; FM—fully methylated; IM—intermediary methylated; CT—chemotherapy; CT-B—chemotherapy with bevacizumab.

Patients were grouped according to their mutation and methylation status. Survival analysis was performed for a group of 68 patients, 18 of whom were long-term survivors with survival of more than 60 months. That group consisted of four *BRCA1/2*-mutated, nine *BRCA1/2*-methylated cases, one with both aberrations, and four *BRCA1/2* mutation- and methylation-free cases (Figure 7a,b). Four out of five mutated cases were treated with PARPi, which may be the possible reason for prolonged survival. We did not observe a significant difference in PFS between the groups (log-rank, *p* = 0.52), nor in the overall survival (log-rank, *p* = 0.15). Stratifying the WT group according to treatment and *BRCA1/2* methylation status, we can observe an unfavorable effect of maintenance treatment with bevacizumab in both of the groups. Median PFS was 37 months for the *BRCA1/2*-mutated group and 26 months for the fully methylated. On the other hand, *BRCA1/2* wild-type tumors with IM promoter median PFS was 22 months, and for patients without any *BRCA1/2* alterations, the median PFS was the shortest (24 months).

Expectedly, in Cox regression univariable analysis, patients with relapsed OC show 2.45 times higher risk of progression compared to newly diagnosed patients, but without reaching statistical significance (*p* = 0.064, HR, 2.45; 95% CI 0.95–6.33). We found no statistically significant impact on progression-free or overall survival in patients for clinicopathological characteristics (FIGO stage, grade, age), nor for the methylation status of *BRCA1* and *BRCA2* genes. Observing treatment approach in the univariable model, we found an unfavorable effect of combination treatment with maintenance bevacizumab and chemotherapy on the overall survival of patients (HR, 2.47; 95% CI 1.16–5.22; *p* = 0.018), compared to chemotherapy only (Figure S6; Supplementary Table S2). The five-year overall survival rate in this cohort was 43.3% (95% CI, 31.1–60.1%). The highest 5-year survival rates were in the *BRCA1* and *BRCA2*-mutated patient groups (50% and 66.7%; 95% CI, 12.5–100%, and 37.9–100%, respectively). The comparison to the *BRCA1/2* WT group did not reach statistical significance (*p* = 0.2 and 0.07, respectively). None of the other *BRCA1/2* aberrations showed a significant difference in survival rate compared to *BRCA1/2* WT. *BRCA1-* and *BRCA2*-methylated patients showed similar 5-year survival rates of 44.6% and 40% (95% CI, 24.9–79.9% and 13.7–100%, respectively). *BRCA* WT cases showed the lowest 5-year survival rate of 35.1% (95% CI, 17–72.4%).

Performing multivariable Cox regression analysis, we adjusted analysis for *BRCA1/2* methylation and *BRCA1/2* mutation status, patient group, type of treatment, and age at diagnosis. Results showed significance only for overall survival in the chemotherapy–bevacizumab combination of therapy (*p* = 0.023; HR, 2.54; 95% CI, 1.14–5.68) (Table 3).

## 4. Discussion

Epigenetic inactivation of *BRCA1/2* genes is postulated to be a marker of response to platinum-based and PARPi systemic therapy by conferring HRD. In this study, we aimed to evaluate the prevalence of *BRCA1/2* promoter methylation in the Serbian population and its association with clinicopathological features, as well as to explore the effects on patient survival.

There is a wide range of reported frequencies of *BRCA1/2* methylation in ovarian tumors in the literature, which may be attributed to population diversity, type of ovarian cancer studied, techniques used for the detection of methylation, and the number of CpG islands covered, as well as the type of material used. For quantitative methods such as quantitative MSP or methylation array, the choice of cut-off methylation level is of particular importance, where some studies have used percentages as low as 5% to call methylation [24,25,26]. Qualitative methods such as MSP or methylation-specific High Melting Resolution (MS-HRM), while lacking a precise methylation level, offer a simple and cost-effective way to determine the presence or absence of methylation and are attractive as complementary tests to *BRCA1/2* mutation testing. The prevalence of homozygous promoter hypermethylation (FM) found in our study cohort (4.1% and 0.45% for *BRCA1* and *BRCA2*, respectively) was comparable to the prevalence of *BRCA1* methylation of 6.4% reported in the US population by Swisher et al. using MSP [24], yet lower than the reported range of 6.4–73.7% for *BRCA1* promoter hypermethylation in OC patients in the meta-analysis conducted by Kalachand et al., where different methods and cut-off values were used to call hypermethylation [24]. Conversely, *BRCA2* methylation is a rather rare event, with different studies reporting diverse prevalence, ranging from none or only one case of *BRCA2* hypermethylation to 21% [21,25,27,28,29,30,31]. Overall *BRCA1/2* dysfunction in this cohort was found in 49.6%, counting *BRCA1/2* hypermethylation (FM and IM cases) and *BRCA1/2* genomic alterations (pathogenic and likely pathogenic variants). Similar results were observed by Kalachand et al., with 40% overall aberrations of *BRCA1/2* in HGSOC [24].

Recent studies demonstrated that the HRD phenotype may also be induced by *BRCA1* promoter methylation, suggesting its use as a predictive biomarker from PARP inhibitor therapy [14,24,31,32,33]. Information about PARPi treatment in *BRCA1*-methylated ovarian cancer is limited, with the ARIEL-2 study reporting a positive effect of methylation on the response to PARPi rucaparib [34]. They demonstrated higher loss of heterozygosity (LOH), which is connected with a higher genomic instability score (GIS) associated with HRD, in one proportion of *BRCA1*-hypermethylated cases [35]. However, studies researching *BRCA2* promoter methylation in response to PARPi are lacking.

An important finding in our cohort was that the patients’ age of diagnosis of ovarian cancer was strongly affected by *BRCA1/2* status. We found similar representation of age in *BRCA1/2*-mutated cases without methylation and *BRCA1/2* wild-type cases which are intermediary methylated (IM). Notably, the group without mutations but with fully methylated *BRCA1/2* had the earliest age of onset, which is in concordance with the finding from a meta-analysis performed by Kalachand et al. [24]. Additionally, none of the methylated cases had previous family history of breast or ovarian cancer, which was observed as well in a study conducted by Baldwin et al. [36]. Hypermethylation is thought to occur almost exclusively in a sporadic manner, suggesting that the methylation of the *BRCA1/2* genes might be employed as a pre-test when the existence of a hereditary nature is suspected [37,38]. Our results confirm reports that *BRCA1* methylation and *BRCA1/2* mutation are mutually exclusive events and that methylation is not likely a “second hit” in Knudson’s two-hit hypothesis [31,34,39,40,41].

While the average *BRCA1* and *BRCA2* expression levels were lower in the FM and IM groups compared to UM samples, the difference was not statistically significant. For the *BRCA2* gene, this result is in concordance with Pradjatmo et al.’s findings [42]. However, other studies demonstrated that *BRCA1* promoter hypermethylation reduces the expression level, contrary to results from our study [24,43,44]. There are several possible explanations for this result that may stem from different levels of methylation in the IM group, technical limitations, or lack of functional effect of probed CpGs. It is important to note that MSP as a technique covers only a limited number of preselected CpG sites, not the entire regulatory region. The presence of a positive event of methylation does not confirm the same for all CpGs in the promoter region. Therefore, the lack of association of expression and methylation can come from the possibility that unscreened CpGs are not methylated, leading to gene expression. Alternatively, the large variation in *BRCA1* and *BRCA2* expression in unmethylated samples could be explained by other epigenetic mechanisms of gene silencing, including closed chromatin modifications, miRNA-mediated translational attenuation or mRNA degradation, or allele-specific expression driven by SNPs, causing a subset of samples in the unmethylated group to show lower levels of gene expression.

Survival analysis in relation to *BRCA1/2* promoter methylation showed no favorable effect on OS or PFS in methylated versus unmethylated subgroups. Prognostic relevance for PFS and OS was not identified in the study performed by Ruscito et al. either [45]. Sahnane reported the opposite results, with a favorable outcome in *BRCA1/2*-methylated patients, as did Swisher et al. in the ARIEL-2 study [25,34]. However, in the further analysis performed by the ARIEL-2 consortium, Kondrashova et al. discovered that the favorable OS effect in the *BRCA1*-methylated group comes from homozygous *BRCA1*-methylated cases. Notably, in the ARIEL-2 study, Kondrashova et al. reported changes from homozygous to heterozygous *BRCA1* methylation status under the pressure of treatment. Since *BRCA1* methylation status can alter over the course of the disease, it may have potential as a biomarker of therapy resistance and a crucial indicator of treatment response in cases of recurrence [46,47]. Regarding the unfavorable effect of combinational treatment with bevacizumab, Fiegl et al. observed the same results [31]. This effect may be due to the criteria for selecting the patients for this type of treatment, as only the patients with higher-stage (FIGO IIIc and IV) or residual disease can be included.

Limitations of this study include the choice of technique for methylation detection, the size of testing cohort, and lack of information about methylation pattern in the complete *BRCA1/2* promoter region after first-line and maintenance treatment in relapsed cases of ovarian cancer patients. Considering the sample size, class imbalance, and the number of events, this study was able to identify only large effect sizes. While we did not observe any large effects on PFS and OS, we cannot rule out moderate or modest survival benefit in *BRCA1/2*-methylated patients. Additionally, our follow-up cohort for survival analysis lacks a group of *BRCA1/2* wt and methylated patients who received PARPi treatment to explore the effect of targeted treatment in this group of patients. MSP was the technique of choice for this study because it is a reproducible and cost-effective technique, that could easily be implemented in clinical practice. However, it is a qualitative method, and we could not obtain the exact percentage of methylation. Furthermore, not all CpG islands could be covered, which may be misleading for some of intermediary and unmethylated samples for both genes. Also, it is important to emphasize that MSP is not a suitable technique for clonal distinction, nor is it a true representation of methylation level through the entire promoter region, as in this technique a few preselected relevant CpGs are screened. Prior to the introduction of HRD as the eligibility criteria for PARPi administration, only relapsed cases with pathogenic variants in either *BRCA1* or *BRCA2* that already responded well to platinum were treated, which excluded this group of patients with hypermethylation. Therefore, a possible predictive role of *BRCA1/2* promoter methylation for PARP inhibitor efficacy could not be evaluated in this cohort due to insufficient treatment and response data.

## 5. Conclusions and Future Outlook

In summary, this study demonstrated that *BRCA1* and *BRCA2* promoter methylation prevalence in Serbian patients is different from that reported in the existing literature. Given the early age of onset in patients with homozygous methylation of either *BRCA1* or *BRCA2*, mutually exclusive with mutation events, and combined with an absence of positive family history, methylation analysis may serve as a pre-test for BRCA1/2 hereditary screening. The large variance observed for *BRCA1/2* expression warrants an investigation of other epigenetic mechanisms as potential mediators of reduced gene expression and HRD phenotype. Finally, prospective studies with longitudinal methylation assessment and standardized treatment response data are necessary to evaluate *BRCA1/2* promoter methylation as a predictive biomarker for PARP inhibitor response.

## Figures and Tables

**Table 1:** Clinicopathological features of patients tested on *BRCA1/2* hypermethylation.

Characteristics		Number	(%)
Age at diagnosis (n = 150)			
	mean	60.84	
	range	17–86	
Age at testing (n = 244)			
	mean	61.99	
	range	17–86	
Patient group (n = 244)			
	Primary carcinoma	103	(42.21)
	Relapse	141	(57.79)
FIGO staging (n = 191)			
	I	10	(5.24)
	II	9	(4.71)
	III	144	(75.39)
	IV	28	(14.66)
Family history (n = 66)			
	ovarian cancer	0	(0.00)
	breast cancer	12	(92.31)
	both	1	(7.69)
Grade (n = 30)			
	G1	1	(3.33)
	G2	11	(36.67)
	G3	16	(53.33)
	G2-3	2	(6.67)
Type of systemic therapy (n = 67)			
	chemotherapy only	28	(41.79)
	chemo and targeted Tx	39	(58.21)
Type of targeted therapy (n = 68)			
	bevacizumab	29	(42.65)
	olaparib	5	(7.35)
	both	5	(7.35)
	no targeted Tx	29	(42.65)
Response to platinum (n = 61)			
	resistant	7	(11.47)
	partial	21	(34.43)
	sensitive	33	(54.1)

**Table 2:** Association between *BRCA1/2* methylation status and clinicopathological factors.

Parameters	BRCA1				BRCA2		
	FM	IM	NM	*p*-Value	IM	NM	*p*-Value
Age at testing							
	*(n = 10)*	*(n = 57)*	*(n = 177)*	*0.07*	*(n = 25)*	*(n = 197)*	*0.02*
mean	55.4	61.12	62.64		58.56	62.82	
range	34–67	35–78	17–86		43–75	17–86	
	
Age at diagnosis							
	*(n = 5)*	*(n = 31)*	*(n = 113)*	*0.062*	*(n = 12)*	*(n = 124)*	*0.026*
mean	52.17	58.42	61.96		55.33	61.99	
range	32–62	35–77	17–86		43–73	17–86	
	
Patient group							
	*(n = 10)*	*(n = 57)*	*(n = 177)*	*0.002*	*(n = 25)*	*(n = 197)*	*<0.001*
Primary carcinoma	1 (10%)	16 (28.07%)	86 (48.59%)		3 (12%)	92 (46.7%)	
Relapse	9 (90%)	41 (71.93%)	91 (51.41%)		22 (88%)	105 (53.3%)	
	
FIGO staging							
	*(n = 9)*	*(n = 47)*	*(n = 135)*	*0.573*	*(n = 20)*	*(n = 150)*	*0.047*
I	1 (11.11%)	2 (4.26%)	7 (5.19%)		2 (10%)	7 (4.67%)	
II	1 (11.11%)	3 (6.38%)	5 (3.70%)		3 (15%)	6 (4%)	
III	6 (66.67%)	37 (78.72%)	101 (74.81%)		11 (55%)	116 (77.33%)	
IV	1 (11.11%)	5 (10.64%)	22 (16.3%)		4 (20%)	21 (14%)	
	
Family history							
	*(n = 5)*	*(n = 16)*	*(n = 45)*	*0.0468*	*(n = 11)*	*(n = 46)*	*0.426*
No	4 (80%)	16 (100%)	33 (73.33%)		10 (90.91%)	35 (76.09%)	
Yes	1 (20%)	0	12 (26.67%)		1 (9.09%)	11 (23.91%)	
Grade							
	*(n = 3)*	*(n = 8)*	*(n = 19)*	*0.288*	*(n = 7)*	*(n = 20)*	*0.560*
G1	1 (33.33%)	0	0		0	0	
G2	0	3 (37.5%)	8 (42.10%)		4 (57.14%)	7 (35%)	
G3	2 (66.67%)	5 (62.5%)	9 (47.37%)		3 (42.86%)	12 (60%)	
G2-3	0	0	2 (10.53%)		0	1 (5%)	
Menopausal status							
	*(n = 5)*	*(n = 17)*	*(n = 49)*	*0.164*	*(n = 11)*	*(n = 50)*	*0.489*
Pre	1 (20%)	3 (17.65%)	6 (12.24%)		1 (9.09%)	8 (16%)	
Peri	2 (40%)	1 (5.88%)	4 (8.16%)		2 (18.18%)	3 (6%)	
Post	2 (40%)	13 (76.47%)	39 (79.6%)		8 (72.73%)	39 (78%)	

**Table 3:** Multivariable Cox regression analysis for overall survival.

	Multivariable PFS			Multivariable OS		
Characteristic	HR	95% CI	*p*-Value	HR	95% CI	*p*-Value
BRCA1/2 methylation status						
BRCA1/2 UM	—	—		—	—	
BRCA1/2 FM	1.58	0.42, 6.02	0.502	1.62	0.49, 5.4	0.432
BRCA1/2 IM	0.96	0.4, 2.29	0.928	0.64	0.27, 1.55	0.322
BRCA1/2 mutation status						
BRCA1/2 WT	—	—		—	—	
BRCA1/2 MUT	1.78	0.37, 8.62	0.472	1.15	0.23, 5.83	0.863
Patient group						
ND	—	—		—	—	
R	2.7	0.96, 7.58	0.059	0.91	0.31, 2.68	0.867
Type of therapy						
CHEMO	—	—		—	—	
CHEMO–Bev	2.19	0.99, 4.84	0.052	2.54	1.14, 5.68	0.023
CHEMO–Bev–PARPi	0.96	0.12, 7.62	0.97	0.37	0.05, 2.9	0.344
CHEMO–PARPi	0.14	0.01, 1.66	0.121	0.27	0.02, 3.09	0.29
Age at diagnosis	1.01	0.97, 1.06	0.62	1	0.95, 1.05	0.929

Abbreviations: CI = confidence interval, HR = hazard ratio. UM—unmethylated; FM—fully methylated; IM—intermediary methylated; WT—wild-type; MUT—mutated; CHEMO—chemotherapy; Bev—bevacizumab; PARPi—poly (ADP-ribose) polymerase inhibitor.

## Data Availability

The data presented in this study are available on request from the corresponding author. The clinical data are available upon request due to ethical considerations and privacy reasons, warranting GDPR compliance and a material transfer agreement with the requesting party.

## Supplementary Materials

The following supporting information can be downloaded at: https://www.mdpi.com/article/10.3390/cells15030277/s1, Figure S1: Bioanalyzer gel electropherogram after methylation-specific PCR; Figure S2: Age at testing and diagnosis by *BRCA1/2* methylation status; Figure S3: Age at testing by *BRCA1* and *BRCA2* aberation status; Figure S4: Level of expression according to overall *BRCA1/2* aberration; Figure S5: Progression-free and overall survival by *BRCA1* and *BRCA2* promoter methylation level; Figure S6: Progression-free and overall survival by treatment; Table S1: Association between BRCA status and clinicopathological factors; Table S2: Univariable Cox regression analysis; Table S3: Comparison of included and excluded patients for survival analysis.

## Abbreviations

The following abbreviations are used in this manuscript:

OC	Ovarian Cancer
EOC	Epithelial Ovarian Cancer
HGSOC	High-Grade Serous Ovarian Cancer
HRD	Homologous Recombination Deficiency
PARPi	Poly (ADP-ribose) Polymerase Inhibitors
LOH	Loss of Heterozygosity
GIS	Genomic Instability Score
OS	Overall Survival
PFS	Progression-Free Survival
PFI	Progression-Free Interval
IORS	Institute for Oncology and Radiology of Serbia
MSP	Methylation-Specific PCR
NGS	Next-Generation Sequencing
TTC%	Tumor Tissue Cell Percentage
FFPE	Formalin-Fixed, Paraffin-Embedded
FM	Fully Methylated
IM	Intermediary Methylated
UM	Fully Unmethylated
BC	Bisulfite Conversion
